# Inter-axonal recognition organizes *Drosophila* olfactory map formation

**DOI:** 10.1038/s41598-019-47924-9

**Published:** 2019-08-09

**Authors:** Gaurav Goyal, Ariane Zierau, Marc Lattemann, Beate Bergkirchner, Dominik Javorski, Rashmit Kaur, Thomas Hummel

**Affiliations:** 1 Department for Neurobiology, University of Vienna, Althanstrasse 14, 1090 Vienna, Austria; 2 Institut für Neuro- und Verhaltensbiologie, Universität Münster, Badestr. 9, D-48149 Münster, Germany

**Keywords:** Axon and dendritic guidance, Molecular neuroscience

## Abstract

Olfactory systems across the animal kingdom show astonishing similarities in their morphological and functional organization. In mouse and *Drosophila*, olfactory sensory neurons are characterized by the selective expression of a single odorant receptor (OR) type and by the OR class-specific connection in the olfactory brain center. Monospecific OR expression in mouse provides each sensory neuron with a unique recognition identity underlying class-specific axon sorting into synaptic glomeruli. Here we show that in *Drosophila*, although OR genes are not involved in sensory neuron connectivity, afferent sorting via OR class-specific recognition defines a central mechanism of odortopic map formation. Sensory neurons mutant for the Ig-domain receptor Dscam converge into ectopic glomeruli with single OR class identity independent of their target cells. Mosaic analysis showed that Dscam prevents premature recognition among sensory axons of the same OR class. Single Dscam isoform expression in projecting axons revealed the importance of Dscam diversity for spatially restricted glomerular convergence. These data support a model in which the precise temporal-spatial regulation of Dscam activity controls class-specific axon sorting thereby indicating convergent evolution of olfactory map formation via self-patterning of sensory neurons.

## Introduction

In mouse and *Drosophila*, olfactory receptor neurons (ORNs) express a single odorant receptor and all neurons of the same OR class converge into distinct synaptic glomeruli^1^. However, different developmental control mechanisms seem to be employed in the formation of these olfactory maps. In mammals, odorant receptors are critical determinants in ORN connectivity by mediating inter-axonal communication^2–5^. A current model proposes that ORs are not directly involved in axon-axon interaction but that OR endogenous activity leads to the expression of a distinct set of cell adhesion molecules^6,7^. This ORN class-specific adhesion code determines local axon sorting and glomerular convergence at a defined position in the olfactory bulb^6,7^. As the postsynaptic target cells are largely dispensable for class-specific ORN axon sorting^8–10^, the formation of an odortopic map in the mouse brain is thought to be controlled by the self-organizing activity of the sensory neurons via the precise regulation of unique axonal recognition identities^11,12^.

In *Drosophila*, the 50 ORN classes in the adult olfactory system show a similar level of sensory and synaptic specificity as in mammals^13^. Each sensory neuron expresses only one type of OR and all neurons of the same OR class converge their axons onto a single synaptic target unit in the brain^14–17^. Inside each of these synaptic glomeruli, ORN axons associate with two main classes of CNS target dendrites, a glomerulus-specific type of relay projection neurons (PNs) and various classes of multi-glomerular local interneurons that mediate signal integration within the first olfactory processing center^18–20^. Despite the similarities in olfactory system organization, OR deletion and misexpression experiments indicate that the *Drosophila* ORs do not have a function in either monospecific receptor expression or the determination of synaptic identity^21–24^. This raises the question if olfactory map formation in *Drosophila* is also organized by an inter-axonal signaling process similar to mammals or if direct axon-dendrite interaction with the target field is the primary patterning mechanism. Previous studies have shown that glomerulus-specific projection neurons (PNs) pre-pattern the target region before afferent arrival suggesting olfactory circuitry formation via direct synaptic partner recognition^13^, though recent studies indicate a more prominent role of interactions between targeting axons in patterning the antennal lobes^25^.

Using genetic approaches, *Drosophila* mutant analyses have identified several cell surface molecules essential for distinct steps in ORN axon targeting^26–30^. The most promising candidate to provide unique recognition identities is the Immunoglobulin (Ig) domain receptor Dscam^31,32^. Alternative splicing of Dscam can generate more than 18,000 distinct recognition molecules that bind in a strictly homophilic manner^33–35^. In nervous system development, Dscam controls the elaboration of axonal and dendritic processes in a cell-autonomous fashion called self-avoidance^36–46^. In the same context, Dscam diversity is required to prevent repulsion between neighboring neuronal processes (non-self association)^36,40,41^. However, no specificity in isoform expression seems to be necessary for neuronal patterning as randomly selected isoforms can substitute for the loss of the endogenous Dscam function^36,37,39–41,46^.

We have shown before that the absence of Dscam causes ORN axons to mistarget into ectopic glomeruli^26^ raising the question of the underlying cellular interactions. Here we demonstrate that afferent sorting via OR class-specific recognition defines a critical mechanism for *Drosophila* olfactory map formation. Loss of Dscam induces premature inter-axonal recognition with ectopic convergence independent of synaptic partner neurons. Our experiments suggest that Dscam, although dispensable for inter-axonal recognition itself, is critical for the precise spatio-temporal regulation of this recognition process. Thus, despite a different requirement of odorant receptors, olfactory map formation in *Drosophila* and mouse is mediated by similar mechanisms of afferent sorting via OR-class specific recognition identities.

## Results

### *Dscam* mutant ORN axons maintain their class-specific recognition identity

**Figure 1:**
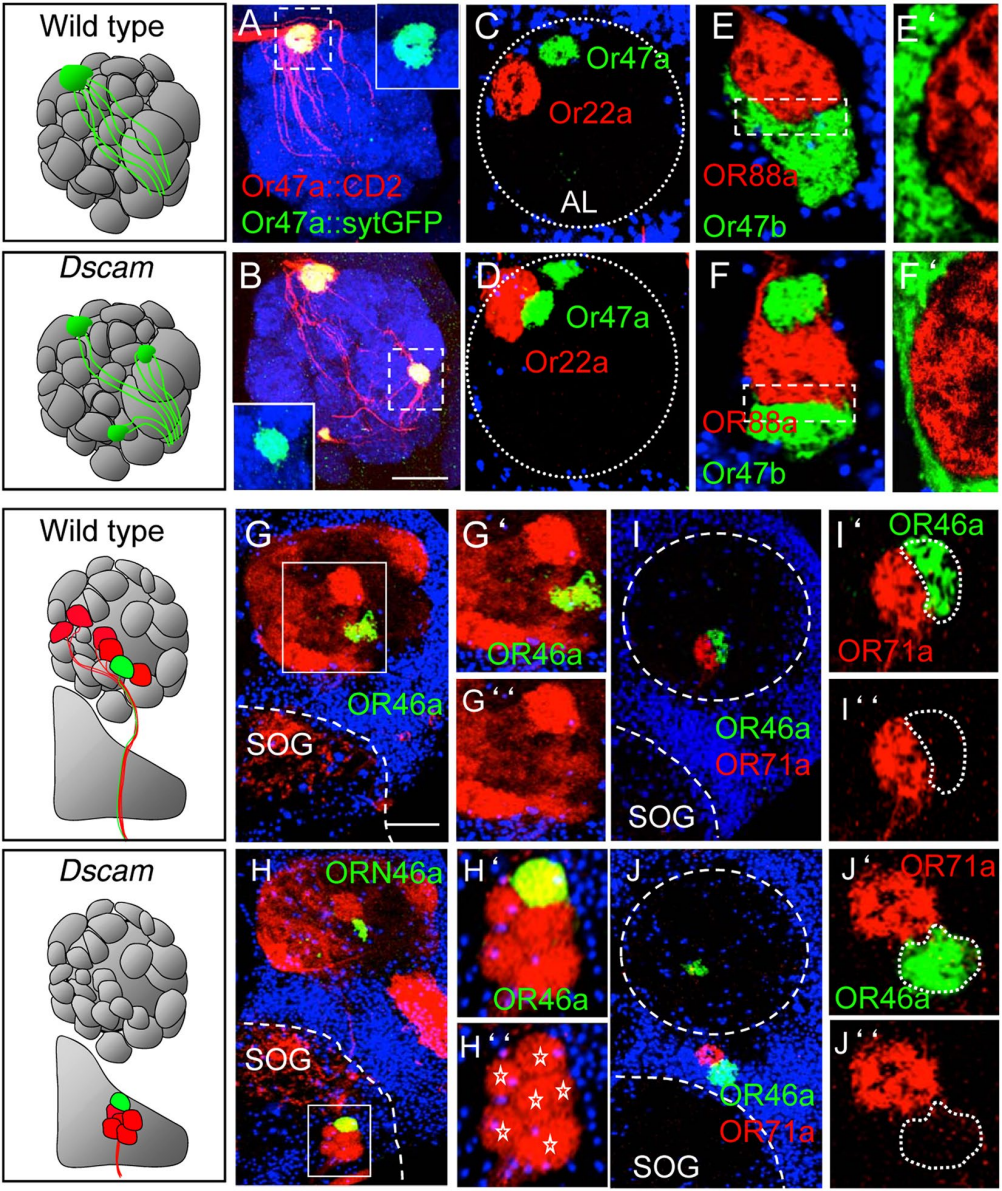
*Dscam* mutant axons converge class-specifically in ectopic spots. (**A**,**B**) Wild type ORN47a axons (red, anti-CD2) grow to their specific target and converge into one glomerulus (green, sytGFP) (**A**) whereas *Dscam* mutant axons converge into an ectopic glomerular-like structure (**B**). (**C**–**J**) Double labelling of different ORN classes show ORN class-specific axon sorting. *Dscam* mutant ORN47a axons converge in the neighbourhood of ORN22a glomerulus but the axons never intermingle (**D**). Neighbouring projecting ORN classes 47b and 88a show distinct boundaries in wild type (**E**,**E**’) as well as in *Dscam* mutants (**F**,**F’**). (**G**,**H**) *Dscam* mutant axons coming from the maxillary palps converge into distinct ectopic spots at the border to the AL or at the suboesophagial ganglion (SOG) (asterisks in **H**”). Ectopic projecting ORN46a axons converge into one distinct spot in the neighborhood of other ectopic spots (**H**’) and they never intermingle with other ORN classes e.g. ORN71a (**J**). SOG: suboesophagial ganglion. Green: (**A**–**J**) sytGFP; blue: (**A**,**B**) N-Cad, (**C**–**J**) Toto3; red: (**A**–**J**) ratCD2. Scale bar: 25 µm. *Genotype:* (***A***) *eyflp UAS-CD2; FRT42 47a::sytGFP/FRT42 PCNA; 47a-Gal4 UAS-CD2*. (**B**) *eyflp UAS-CD2; FRT42 Dscam 47a::sytGFP/FRT42 PCNA; 47a-Gal4 UAS-CD2*. (**C**) *eyflp UAS-CD2; FRT42 47a::sytGFP/FRT42 PCNA; 22a-Gal4 UAS-CD2*. (**D**) *eyflp UAS-CD2; FRT42 Dscam 47a::sytGFP/FRT42 PCNA; 22a-Gal4 UAS-CD2*. (**E**) *eyflp UAS-CD2; FRT42 47b::sytGFP/FRT42 PCNA; 88a-Gal4 UAS-CD2*. (**F**) *eyflp UAS-CD2; FRT42 Dscam 47b::sytGFP/FRT42 PCNA; 88a-Gal4 UAS-CD2*. (**G**) *eyflp; FRT42/FRT42 PCNA; 46a::sytGFP/MT14-Gal4 UAS-CD2*. (**H**) *eyflp; FRT42 Dscam/FRT42 PCNA; 46a::sytGFP/MT14-Gal4 UAS-CD2* (**I**) *eyflp UAS-CD2; FRT42/FRT42 PCNA; 46a::sytGFP 71a-Gal4 UAS-CD2*. (**J**) *eyflp UAS-CD2; FRT42 Dscam/FRT42 PCNA; 46a::sytGFP 71a-Gal4 UAS-CD2*.

In the adult *Drosophila* olfactory system, about 1500 ORNs located in peripheral epithelia subdivide into 50 functional classes according to the OR expression^13^. Each sensory class consists of an average of 20–30 ORNs which converge their axons to a single synaptic glomerulus on the ipsi-lateral antennal lobe (AL; Fig. 1A,C,E)^13^. Due to this OR monospecificity, each of the 50 glomeruli in the antennal lobe contains axons of only a single OR class thereby providing the morphological basis of odor recognition^13^. To understand the role of Dscam in OR monospecificity, we generated *Dscam* mutant clones specifically in ORN precursors of the antennal disc using *eyless-flp*^47^. To increase the number of homozygous mutant ORNs, the Dscam^+^ chromosome contained a cell lethal *PCNA* allele^48^ (Methods). In these ORN specific *Dscam* mosaics, ORN axons project into additional glomerulus-like structures^26^ (Fig. 1B,D,F). This mutant axon coalescence can be in close neighborhood to the cognate target glomerulus (“glomerular split”, e.g. Fig. 1D,F) or more distant to the target region inside or even outside the antennal lobe (“ectopic glomeruli”, e.g. Fig. 1B).

We first addressed the question if these additional glomeruli maintain afferent OR monospecificity or contain axons of multiple ORN classes. Local convergence defects were analyzed by visualizing the projection of ORN class pairs that target two neighboring glomeruli (Fig. 1D,F, Supplementary Fig. S1). In all ORN specific *Dscam* mosaics with large mutant clones that show a local rearrangement of the glomerular position (ORN47a/22a n = 14; ORN47b/88a n = 22), axons of different ORN classes do not intermingle but segregate according to the OR identity. To determine the OR class identity of distant ectopic glomeruli we combined a broad ORN marker and a non-overlapping single OR class marker (Supplementary Fig. S2). In all of the analyzed ORN specific *Dscam* mutant mosaic brains (*Con* > *CD2/OR:sytGFP*, n > 10 per OR class; *MT14* > *CD2/OR:sytGFP*, n = 10 per OR class) ectopic glomeruli in different regions of the antennal lobe target area maintain their single ORN-class identity. From these data we conclude that *Dscam* mutant ORN axons are still able to sort out according to their sensory class identity.

### Class-specific axon coalescence is independent of target neurons

To determine if OR class specific axon sorting is mediated through a direct interaction with their target cells we analyzed *Dscam* mutant maxillary ORNs, which stop frequently outside the AL target area into multiple glomerulus-like structures^26^ (Fig. 1G–J). These clones were again generated using ORN specific *eyless-flp* with cell lethal *PCNA* allele on *Dscam*^+^ chromosome to generate large clones (Methods). First, we combined a general marker line including all of the six maxillary ORN classes together with an ORN class-specific marker (Fig. 1G). In *Dscam* ORN mosaic brains, we frequently observed a cluster of up to six ectopic glomeruli ventrally to the AL, in which all ORN axons of the same OR identity are confined to a single glomerulus (n = 15, Fig. 1H). Second, we labeled different pairs of maxillary ORN classes, which in wild type project into neighboring or more distant glomeruli ((Fig. 1I, Supplementary Fig. S3). Similar to ectopic coalescence inside the AL, *Dscam* mutant ORN axons that coalesce outside the AL target area segregate strictly according to their OR class identity (Fig. 1J, n > 30, Supplementary Fig. S3). As these ectopic glomeruli do not contain dendrites of projection neurons (PNs) (see below), we conclude that direct inter-axonal signaling is a critical component of ORN axons sorting.

### Local interneurons but not projection neurons innervate ectopic glomeruli

**Figure 2:**
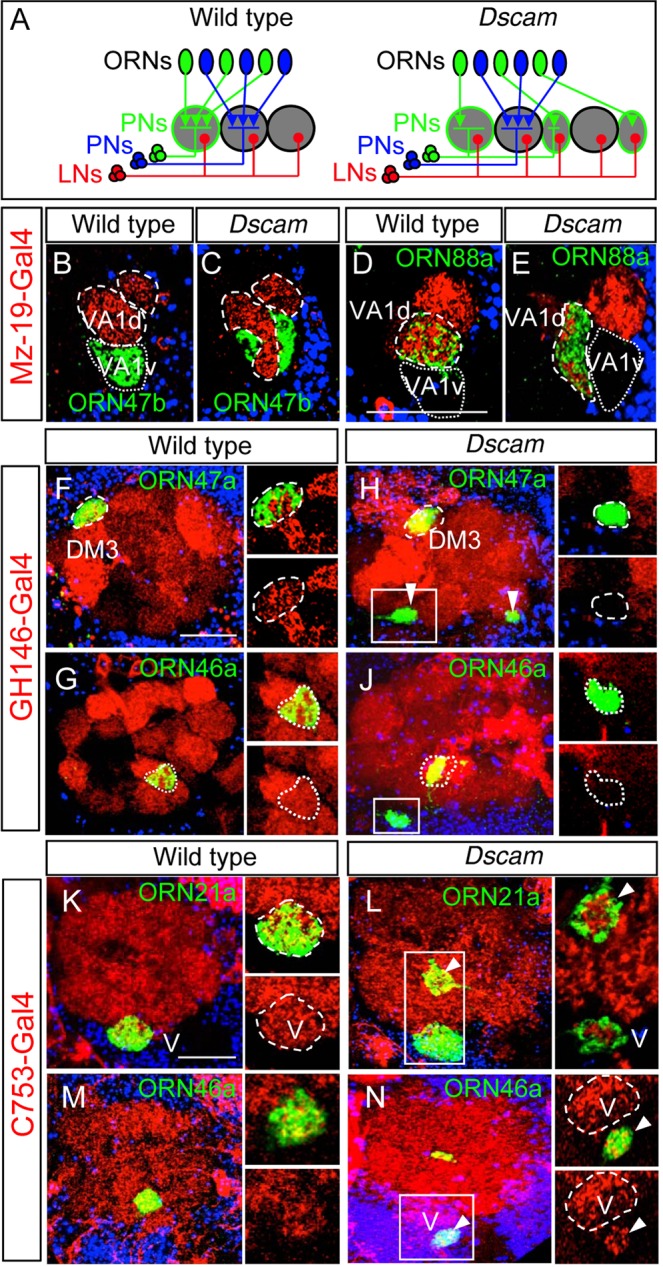
Pre- and postsynaptic recognition is Dscam independent. (**A**) Schematic showing ORN/PN/LN matching in olfactory lobes of *Drosophila* in wild type and *Dscam* mutants. (**B**–**E**) ORN-PN matching identities remain in *Dscam* mutants. Mz-19 positive PN dendrites connect to axons of ORN class 88a (**D**) and not to 47b (**B**) in wild type. In *Dscam* mutant, the dendrites follow the misprojecting 88a axons (**E**) but avoid ectopic 47b axons (**C**). (**F**–**J**) *GH146* expressing dendrites do not innervate ectopic *Dscam* mutant ORN 47a spots, when they are far away from the wild type glomerulus (box in **H**, compared to **F**). Ectopic spots of ORN46a axons outside of the AL are not innervated by *GH146*-positive dendrites (**G**,**J**) even if the glomerulus of the ORN46a class is innervated by *GH146*-positive dendrites. (**K**–**N**) Ectopic *Dscam* mutant spots are innervated from C753-positive LNs in the AL in case of ORN21a (**L**) as well as outside the AL in case of ORN46a (**N**). Green: sytGFP, red: ratCD2, blue: Toto3. Scale bar: 25 µm. *Genotype:* (**B**) *eyflp UAS-CD2; FRT42/FRT42 PCNA; 47b::sytGFP Mz19-Gal4 UAS-CD2*. (**C**) *eyflp UAS-CD2; FRT42 Dscam/FRT42 PCNA; 47b::sytGFP Mz19-Gal4 UAS-CD2*. (**D**) *eyflp UAS-CD2; FRT42/FRT42 PCNA; 88a::sytGFP Mz19-Gal4 UAS-CD2*. (**E**) *eyflp UAS-CD2; FRT42 Dscam/FRT42 PCNA; 88a::sytGFP Mz19-Gal4 UAS-CD2*. (**F**–**G**) *eyflp UAS-CD2; FRT42 OR::sytGFP/FRT42 PCNA; GH146-Gal4 UAS-CD2*. (**H**–**J**) *eyflp UAS-CD2; FRT42 Dscam OR::sytGFP/FRT42 PCNA; GH146-Gal4 UAS-CD2*. (**K**,**M**) *eyflp UAS-CD2; FRT42 OR::sytGFP/FRT42 PCNA; C753-Gal4 UAS-CD2*. (**L,N**) *eyflp UAS-CD2; FRT42 Dscam OR::sytGFP/FRT42 PCNA; C753-Gal4 UAS-CD2*.

In wild type *Drosophila*, the class-specific coalescence of ORN axons into distinct glomeruli is matched by a similar level of class-specific PN dendrite innervation^18,49^. As Dscam does not mediate ORN recognition identity, we asked if it might be involved in axon-dendrite matching. To test this, we generated ORN specific large *Dscam* mutant clones using *eyless-flp/PCNA*, thus PNs and LNs were *Dscam*^+*/−*^. In wild type (no Dscam mutation in the background), ORN88a axons connect to *Mz19*-positive PNs^50^. The removal of *Dscam* from ORNs leads to the local reorganization of the glomerular field, in which ORN88a glomeruli are often displaced by neighboring ORN47b axons (compare Fig. 1E,F). In all analyzed *Dscam* mosaic brains (n > 10), the changes in glomerulus localization lead to a corresponding shift in the PN dendritic field, ensuring that the class-specific ORN-PN matching is maintained (Fig. 2B–E). For more distant ectopic glomeruli we characterized the innervation of *Dscam* mutant ORN axons with *GH146*-positive PN dendrites, which cover all regions of the AL. The ORN classes 21a and 47a, which are not innervated by *GH146*-positive PNs in wild type, form ectopic glomeruli in *Dscam* mosaics but do not receive *GH146*-innervation (Fig. 2F,H, Supplementary Fig. S4; n > 40). In addition, the *Dscam* mutant maxillary ORN 46a that converge outside the AL are never associated with *GH146*-positive PN dendrites although in wild type this ORN class connects to *GH146*-PNs (Fig. 2G,J; n = 6).

On the contrary, removal of Dscam in PNs using MARCM^51^ (using *hs-flp* to generate small PN clones, Supplementary Fig. S5) and targeted *Dscam*-RNAi expression (Using PN specific *GH146-Gal4*, Supplementary Fig. S6) does not affect axonal convergence of wild type ORNs (Supplementary Figs S5and S6; n = 21 for *Dscam* MARCM clones; n = 10 for *GH146* > *Dscam*^*RNAi*^ per OR class).

To test if ectopic glomeruli are accessible for postsynaptic neurites we analyzed their interaction with local interneurons (LNs). In the wild type AL, LNs do not display glomerulus-specificity but elaborate their neurites throughout the AL (Fig. 2K,M). In ORN-specific large *Dscam* mosaic brains, each of the ectopic glomeruli inside the AL receives postsynaptic innervation from LNs (n = 20 per OR class), indicating that the distant ectopic ORN axons are able to interact with the neurites. Furthermore, even the *Dscam* mutant ectopic glomeruli that are formed adjacent to the AL attracts LN neurites (Fig. 2K–N; n = 8).

In summary, *Dscam* mutant glomeruli localized close to the target area receive the correct postsynaptic innervation. During wild type ORN convergence, PN dendrites occupy broad AL region and restrict subsequently onto a single glomerulus, suggesting that locally displaced protoglomeruli in *Dscam* mosaics are in contact with the dendrites of their PN partners^13^. In contrast, distant ectopic glomeruli do not associate with PN dendrites indicating a high level of ORN-PN recognition specificity in the *Drosophila* olfactory system, which is not affected in *Dscam* mutant ORNs. On the other hand, LN neurites seem to associate with all protoglomeruli, which have been formed within their range independent of the ORN class identity. As Dscam does not seem to affect the interaction of ORN axons with their target neurites we further characterized its role in the inter-axonal recognition.

### Formation of ectopic glomeruli requires inter-axonal recognition

**Figure 3:**
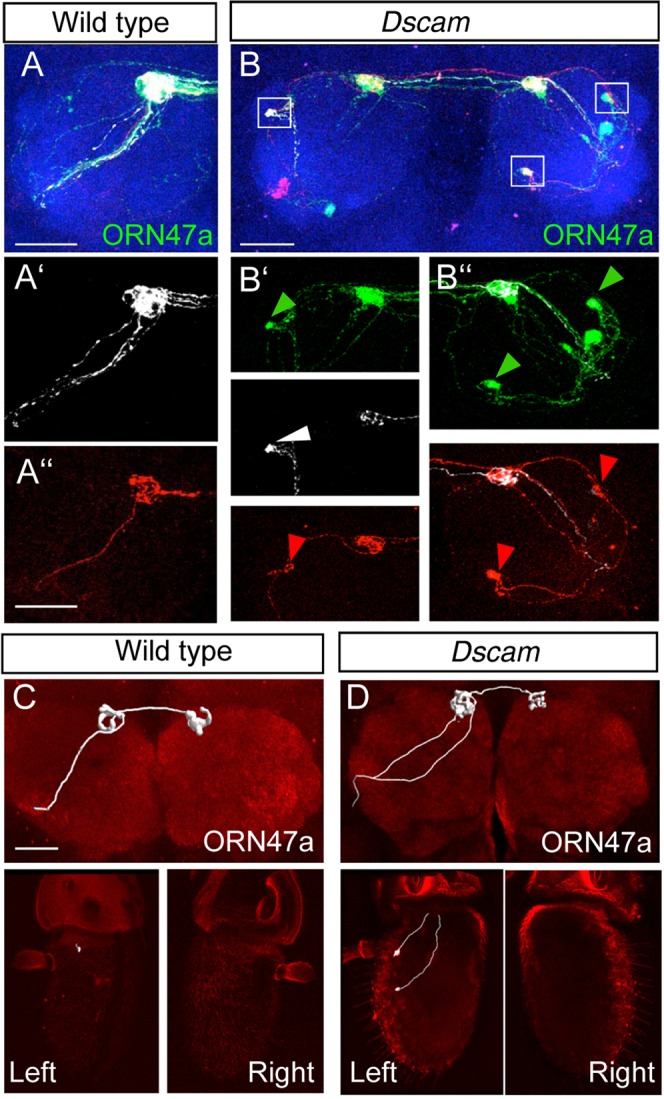
Formation of ectopic glomeruli requires inter-axonal recognition. **(A**,**B**) Multicolour axon labeling using flybow shows targeting of individual mutant ORN 47a axons in WT (**A**-A”) and Dscam (**B**-B”) mutants. The ectopic glomeruli in *Dscam* mutants always consisted of multiple axons (marked with arrow heads in B’ and B”). **(C**,**D**) Visualizing targeting of single/few cell OR47a clones in WT (**C**) and *Dscam* mutants (**D**) showed similar phenotype with OR47a axons not mis-targetting in *Dscam* mutants. The number of ORNs was confirmed by visualizing cell bodies in the left and right antenna. Blue: Ncad (**A**,**B**), Red: Ncad (**C**,**D**). Scale bar: 25 µm. *Genotype:* (**A**) *eyflp; FRT42/FRT42 PCNA; UAS-FB1*.*1*, *Or47a-Gal4/hs-mflp5* (**B**) *eyflp; FRT42 dscam*^*21*^*/FRT42 PCNA; UAS-FB1*.*1*, *Or47a-Gal4/hs-mflp5* (**C**) *eyflp; FRT42/FRT42 PCNA; UAS-FB1*.*1*, *Or47a-Gal4/hs-mflp5* (**D**) *hsflp; FRT42 dscam*^*21*^*/FRT42 Gal80; Or47a-Gal4*, *UAS-mCD8::GFP*.

To determine if inter-axonal recognition is required for the formation of ectopic glomeruli, we analyzed the projections of single *Dscam* mutant ORNs surrounded by other *Dscam* mutant or wild type ORNs. First, we used flybow^52,53^ to visualize individual mutant Or47a axons in the background of a large ORN specific *Dscam* mutant field (generated using *eyeless-flp/PCNA*). Visualizing individual *Dscam* mutant ORN47a axons showed that even small ectopic glomeruli consist of multiple axons, which indicates inter-axonal recognition as a possible mechanism for ectopic axon convergence (Fig. 3A,B). Next we visualized axon targeting of small *Dscam* mutant clones in a Dscam heterozygous mutant field using MARCM. This showed that single *Dscam* mutant ORNs could target normally to the wild type site (Fig. 3C,D, 46/48 1–3 cell clones show WT targetting). This observation combined with results from Flybow clones and convergence of mutant maxillary ORNs outside antennal lobe (above) showed that formation of ectopic glomeruli is a result of interactions between axons of the same OR class.

### Dscam functions in a cell autonomous manner

**Figure 4:**
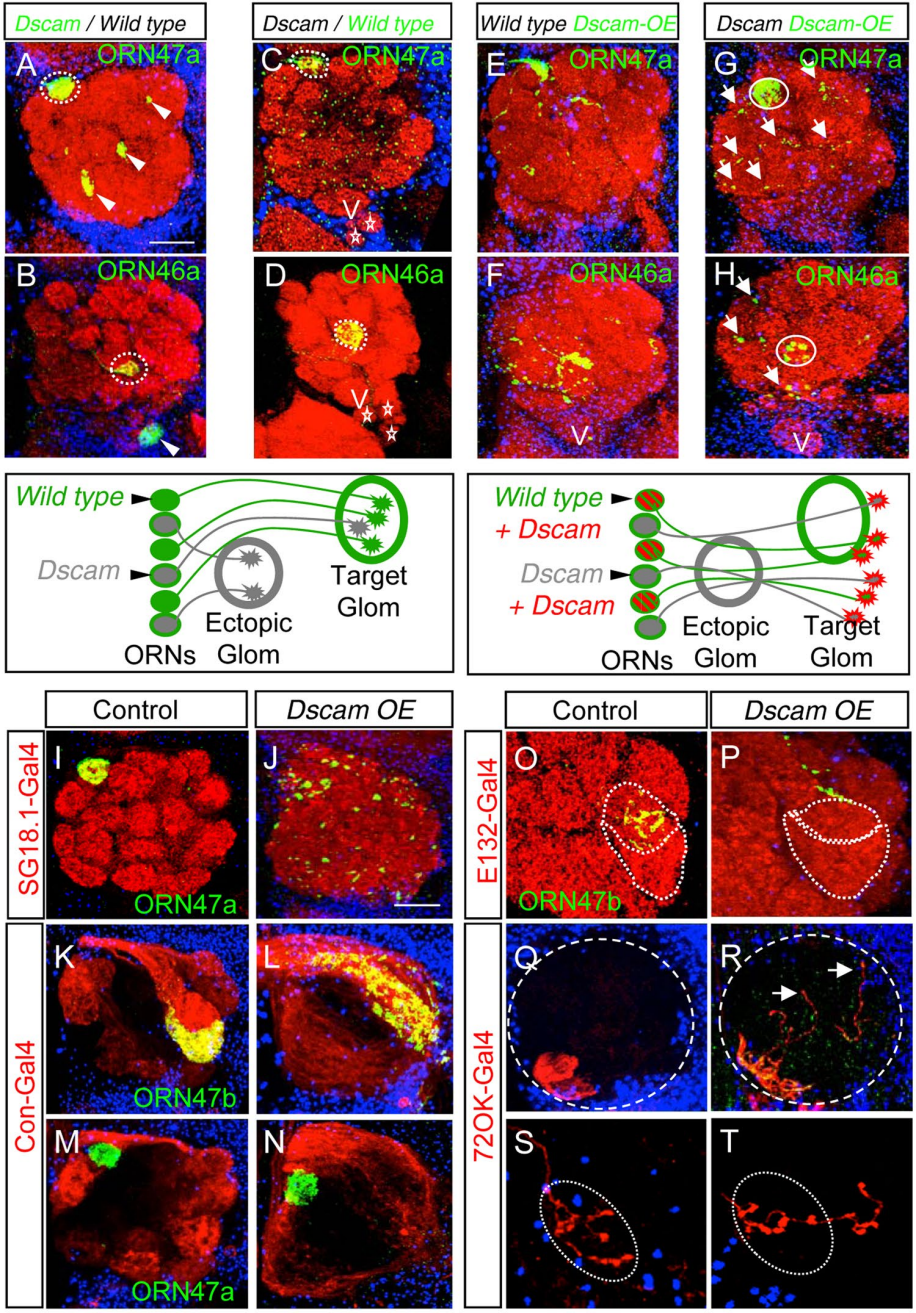
Dscam acts cell-autonomously. (**A**–**H**) Ectopic spots inside (**A**, arrow heads) as well as outside (**B**, arrow head) the AL are innervated only from homozygous *Dscam* mutant axons, whereas homozygous wild type axons always reach their wild type glomerulus (**C**,**D**, dotted circle). Presence of *Dscam* mutant axons is indicated by the presence of ectopic spots outside the AL, near the V glomerulus (asterisks). Over-expression of one single Dscam-isoform in wild type axons leads to disrupted glomerular pattern and axon termini are spread over a large area (**E**,**F**). Over-expression in *Dscam* mutant axons shows the same phenotype than the over-expression in wild type (**G**,**H**). Only the early stopping phenotype outside the AL of maxillary ORN axons can be rescued by over-expression of a single Dscam-isoform (**H**). (**I**–**T**) Broad over-expression of a single Dscam-isoform in ORNs show an AL wide distribution of axon termini (green in J) and a complete loss of the glomerular structure (red in J). Dscam over-expression affects only ORN axons, in which it is expressed. On over-expression in the con-positive ORNs, the structure of the glomerulus innervated from ORN class 47b is totally disrupted (**L**) whereas the ORN class 47a, which project neighboring to the con-positive domain, is unaffected (**N**). Over-expression of a single Dscam isoform in single ORN axons also reveal a misprojecting phenotype (**P**,**R**,**T**). Green: sytGFP, blue: Toto3, red: (**A**–**J**,**O**,**P**) N-cad, (**K**–**N**,**Q**–**T**) ratCD2. Scale bar: 25 µm. *Genotype:* (**A**,**B**) *eyflp; FRT42 Dscam/FRT42 Gal80; OR-Gal4 UAS-sytGFP*. (**C**,**D**) *eyflp; FRT42 Dscam Gal80/FRT42; OR-Gal4 UAS-sytGFP*. (**E**,**F**) *eyflp elav-Gal4; FRT42 OR::sytGFP/FRT42 Gal80; UAS-Dscam*^*17*.*2-7*^. (**G**,**H**) *eyflp elav-Gal4; FRT42 Dscam OR::sytGFP/FRT42 Gal80; UAS-Dscam*^*17*.*2-7*^. (**I**) *SG18*.*1-Gal4*/*OR::sytGFP*. (**J**) *SG18*.*1-Gal4/OR::sytGFP; UAS-Dscam*^*17*.*2-7*^. (**K**,**M**) *OR::sytGFP; con-Gal4 UAS-CD2*. (**L**,**N**) *OR::sytGFP; con-Gal4 UAS-CD2/UAS-Dscam*^*17*.*2-7*^, (**O**) *hsflp E132-Gal4 UAS-sytGFP; FRT42 Gal80/FRT42*. (**P**) *hsflp E132-Gal4 UAS-sytGFP; FRT42 Gal80/FRT42;UAS-Dscam*^*17*.*2-7*^. (**Q**) *OK72-Gal4 UAS-CD2*, (**R**) *eyflp; FRT42 OK72-Gal4 UAS-CD2/FRT42 Gal80; UAS-Dscam*^*17*.*2-7*^, (**S**) *hsflp; FRT42 OK72-Gal4 UAS-CD2/FRT42 Gal80*. (**T**) *hsflp; FRT42 OK72-Gal4 UAS-CD2/FRT42 Gal80; UAS-Dscam*^*17*.*2-7*^.

To determine how Dscam modulates self-recognition of axons from the same OR class we performed a series of mosaic and rescue experiments. First, are wild type axons in genetic mosaics also attracted to coalesce into *Dscam* mutant ectopic glomeruli? The selective labeling of the homozygous wild type axons in “reverse MARCM” clones^54^ revealed that they bypass the ectopic glomeruli and project to their wild type target glomerulus (Fig. 4A–D, Supplementary Fig. S7, n(ORN47a) = 12, n(ORN46a) = 6, n(ORN47b) = 8, n(ORN21a) = 12). This result indicated that *Dscam* functions in a cell-autonomous manner to prevent inter-axonal recognition. Interestingly, some of the *Dscam* mutant axons also reach the WT target site (Fig. 4A,B). Second, we tested if the expression of a single Dscam isoform in mutant ORNs could rescue the ectopic glomerulus formation. An early developmental expression (using *elav-Gal4*) of different Dscam isoform (e.g. *Dscam*^*1*.*30*.*30*.*2*^ *or Dscam*^*11*.*31*.*25*.*2*^) in *Dscam* mutant ORNs (generated using *eyless-flp*) suppresses the ectopic convergence inside and outside the AL (Fig. 4G,H). However, we also observed a disruption of ORN convergence at the target side and axons bypass their target glomerulus (See also Fig. 4R,T). Interestingly, we observed aggregates of Synaptotagmin::GFP along the axonal lengths (Fig. 4G arrows). As a similar axon targeting defect was observed following the expression of the same Dscam isoform in wild type ORNs (Fig. 4E,F), we conclude that the predominant expression of a single Dscam isoform prevents ORN axon convergence. These results suggest that the precise level or isoform diversity of Dscam is necessary to allow correct olfactory system patterning.

### Over-expression of a single Dscam isoform blocks axon convergence

To learn more about the repulsive activity of Dscam in ORN axon convergence, we performed a series of cell-type specific Dscam over-expression experiments. First, single Dscam isoform expression in all olfactory sensory neurons (using *SG18*.*1-Gal4*) completely disrupts axon convergence leading to an aglomerular AL target field (Fig. 4I,J). Second, Dscam over-expression in ORN subgroups, which target to distinct regions within the AL (*Connectin-gal4* expresses in ORN classes projecting onto the lateral antennal lobe), prevents only the convergence within the expression domain whereas adjacent glomeruli are not affected (Fig. 4K–N, Supplementary Fig. S8). Third, if we express Dscam early in development (using *E132* or *72OK-Gal4*) in single (using *hs-flp* generated clones) or all neurons of an individual ORN class, we observed a normal projection towards their target region but a failure to coalesce (Fig. 4O–T). These results suggest that the expression of identical isoforms on neighboring ORNs does not affect their projection towards the target area but neurons do not coalesce into a single glomerulus.

**Figure 5:**
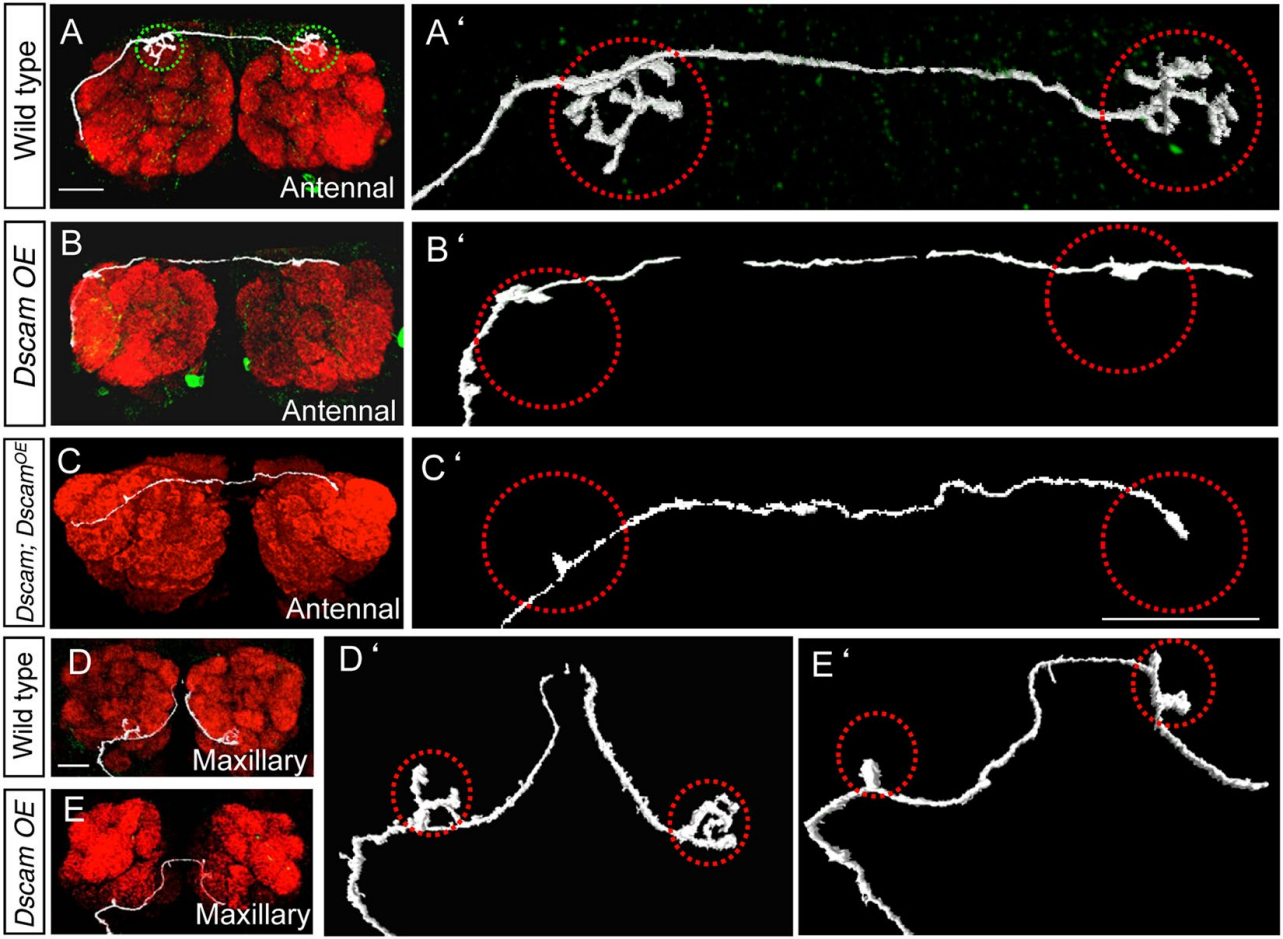
Over-expression of single Dscam isoform prevents targeting in a single neuron. (**A**,**D**) Single labelled wild type ORN axons from the antenna (**A**) and the maxillary palp (**D**) showing branching inside one glomerulus (**A’,D’**) and the contralateral branch. (**B**,**C**,**E**) Over-expression of a single Dscam isoform leads to defective branching inside the WT glomerulus (**B’,E’**) as well as *Dscam* mutant (**C’**). White: mCD8::GFP, Red: N-cad. Scale bar: 25 µm. *Genotype:* (**A**,**D**) *hsflp*, *elav-Gal4 UAS-mCD8::GFP; FRT42 Gal80/FRT42*. (**B**,**E**) *hsflp*, *elav-Gal4 UAS-mCD8::GFP; FRT42 Gal80/FRT42; UAS-Dscam*^*17*.*2-7*^. (**C**) *hsflp*, *elav-Gal4 UAS-mCD8::GFP; FRT42 Gal80/FRT42 Dscam; UAS-Dscam*^*17*.*2-7*^.

The fact that wild type ORN axons that are in contact with Dscam over-expressing axons are not affected in their projection pattern suggests that the Dscam-induced axon repulsion is mediated through homophilic isoform recognition between interacting ORN axons. This would predict that Dscam expression in a single ORN will not interfere with the axon projection as shown before for mushroom body^55^. However, we observed a similar axon overshoot phenotype by expressing Dscam in a single ORN of different classes (Fig. 5A–E) in otherwise WT background. A more detailed single axon analysis showed that the terminal arborization of wild type axons is strongly reduced in Dscam expressing ORNs (Fig. 5B,C,E). In addition, small collateral extension can be observed in irregular positions along the axon shaft.

Thus, in agreement with the cell-autonomous role of Dscam observed in loss-of-function studies, these gain-of-function experiments also support a mechanism of axon convergence in which Dscam does not mediate inter-axonal communication directly but Dscam activity on projecting axons suppresses axonal convergence among ORNs of the same sensory class.

### Regulation of ORN axon convergence during development

**Figure 6:**
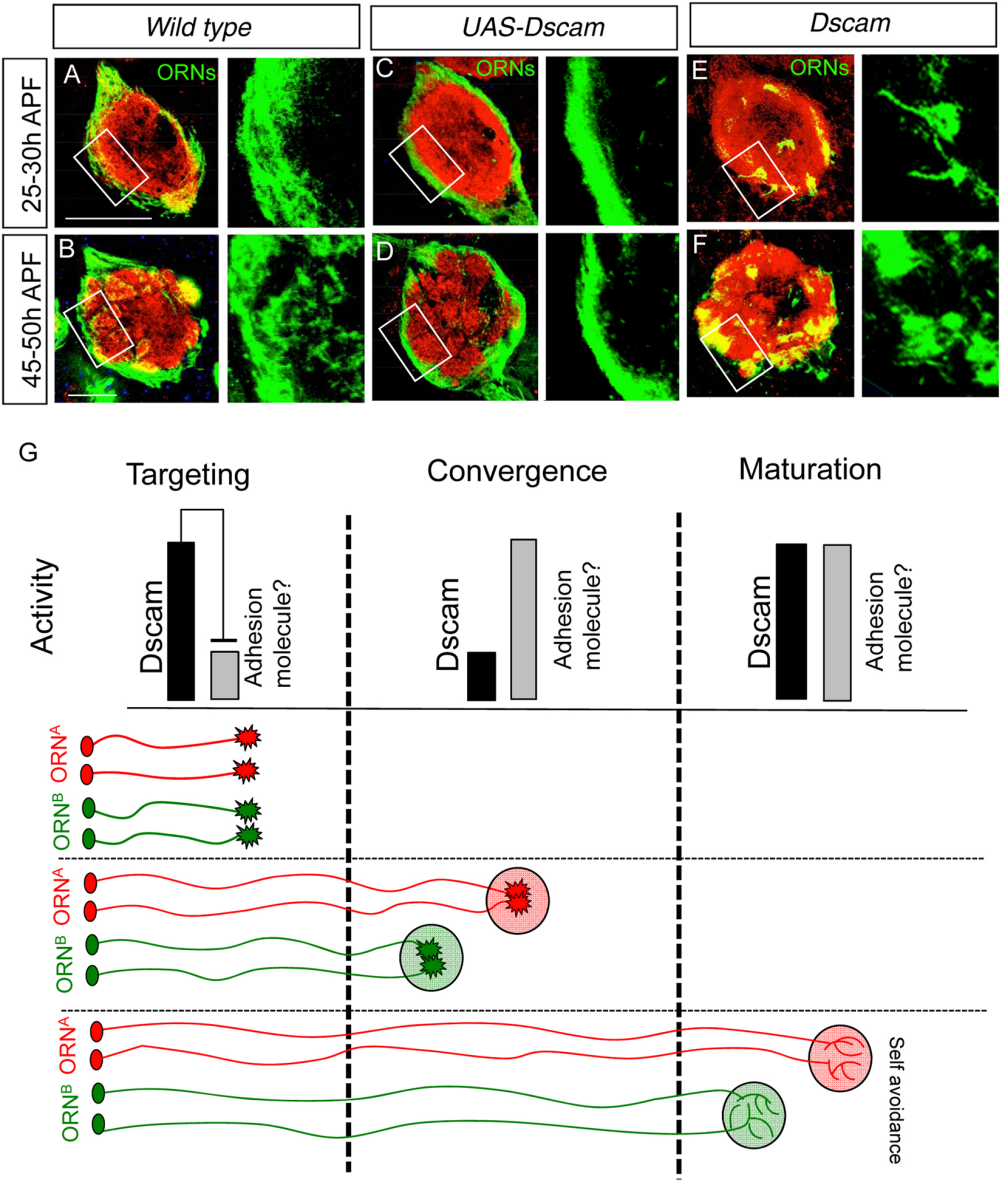
Dscam function is needed in early pupal development. At 25–30 h APF (after puparium formation) the ORN axons surround the developing AL before growing into the center(**A**). Over-expression of a single Dscam isoform leads to a compaction of the surrounding axon tract (**C**) whereas in *Dscam* mutants the ectopic pre-stopping and ectopic convergence is already visible (**E**). During 45–50 h APF in wild type the axons grow into the central part of the AL and start the glomerulus formation (**B**), over-expression of Dscam prevents the growth of the axons into the central part of the AL (**D**). In *Dscam* mutants the amount of ectopic spots increases (**F**). Green: mCD8::GFP; red: N-cad. Scale bar: 25 µm. *Genotype:* (**A**,**B**) *eyflp; FRT42/FRT42 Gal80; elav-Gal4 UAS-mCD8::GFP*. (**C**,**D**) *eyflp; FRT42 UAS-Dscam*^*17*.*2-1*^*/FRT42 Gal80; elav-Gal4 UAS-mCD8::GFP*. (**E**,**F**) *eyflp; FRT42 Dscam/FRT42 Gal80; elav-Gal4 UAS-mCD8::GFP*.

Finally, we determined the developmental step at which Dscam controls ORN axon recognition and convergence. We generated ORN specific *eyless-flp* clones and used *elav-Gal4* for early developmental expression and labeling of ORNs. In wild type, ORN axons project along the periphery of the AL (Fig. 6A, n > 10) where they coalesce into class-specific protoglomeruli before they merge with the dendritic field (Fig. 6B, n > 10). The over-expression of a single Dscam isoform in ingrowing ORN axons fully prevents their initial coalescence into protoglomeruli (Fig. 6C,D, Supplementary Fig. S9, n > 10). These axons stay in the nerve fiber layer and never interact with the dendritic field. In contrast, ORN axons mutant for *Dscam* coalesce into protoglomeruli as soon as they enter the target area thereby preventing their projection along the AL surface (Fig. 6E, n > 10). The number and size of these protoglomeruli increases during further AL development (Fig. 6F, n > 10) but these accumulations of axon terminals fail to interact with the dendritic field. These results show that Dscam is critical in the initial control of ORN axon convergence. The Dscam activity has to be tightly regulated to guarantee a spatially fixed axon convergence as a reduction in Dscam leads to premature axon recognition whereas an increase in Dscam activity prevents axons convergence at the target area.

## Discussion

Sensory maps in the olfactory system are characterized by an astonishing level of synaptic specificity. A multitude of sensory neuron classes, broadly distributed in the peripheral epithelium, project into the CNS primary target region where they segregate into distinct synaptic glomeruli according to their OR class identity. In the mouse olfactory system, the odorant receptors, expressed on the projecting ORN axons, are thought to provide unique recognition identities for the ORN class specific axon sorting in the olfactory bulb^2–5^. However, ORs are not directly involved in the inter-axonal recognition but determine the expression of a distinct combination of cell surface receptors and adhesion molecules that provide spatial information and unique axonal specificity^6,7^.

It has been shown before that Ig-domain adhesion molecule Dscam plays a critical role in olfactory map formation in *Drosophila*^26^. *Dscam* mutant ORNs frequently mistarget and form ectopic glomeruli^26^. On the other hand, Dscam is not involved in synaptic recognition between presynaptic ORNs and post-synaptic PNs^45^. Here we show that although ORs are not involved in sensory neuron connectivity, ORN axons also carry unique identity tags for ORN class-specific recognition independent of their target area. Dscam is critical for the precise regulation of inter-axonal recognition but does not provide recognition identity itself. Loss of *Dscam* leads to class-specific convergence before ORN axons reach their target area. In contrast, Dscam over-expression in wild type suppresses the ORN axon convergence at ectopic sites as well as at the presumptive target area. Overall, Dscam mediated inhibition of inter-axonal recognition in developing ORNs of same OR class seems to be controlled by a cell intrinsic mechanism (similar to Dscam mediated self-avoidance) instead of inter-axonal binding of Dscam between ORN axons of same OR class.

From these data we propose a model of how a balanced Dscam activity controls spatially precise ORN convergence (Fig. 6G). Dscam expression on projecting ORN axons prevents the premature recognition between axons of the same OR class which would lead to the coalescence into protoglomeruli. The Dscam activity has to be overcome as ORN axons enter the target area to allow axon coalescence into OR class-specific protoglomeruli. The signal is most likely derived from the target field as PN dendrites are known to build a pre-patterned dendritic field before ORN axon arrival^18^. The predicted spatial cues could function through the down-regulation of Dscam signaling or enforce inter-axonal adhesion independent of Dscam^31,42,43,56–61^. Later self-recognition between identical Dscam isoforms expressed on individual ORs ensures proper glomerular maturation and synaptogenesis.

### The role of Dscam diversity in olfactory system development

The broad expression of a single Dscam isoform in wild type and *Dscam* mutant ORNs allows a normal projection towards their target area but disrupts axon convergence at the prospective target side. This indicates that Dscam isoform diversity is important to prevent inter-axonal repulsion, which would interfere with the target-induced axon convergence. Here, inter-axonal Dscam binding and recognition is prevented through the expression of non-overlapping sets of Dscam isoforms. The phenotype described in our Gal4-driven over-expression studies resembles the olfactory connectivity defect observed in transgenically engineered flies that express only a single Dscam isoform in all neurons^46^. As we observed the same axonal phenotype with Gal4 lines of different expression strength, increased Dscam signaling due to an enhanced homophilic isoform binding is most likely the cause of the induced axon targeting defects. The lack of early ORN class specific marker lines has prevented the identification of the Dscam isoform expression profile in distinct sensory neurons classes so far. However, based on earlier expression analysis^26^ and identification of isoform expression profiles from other neuronal cell types^37,62^ suggests that each ORN expresses a unique set of Dscam isoforms, which could be more similar among members of one ORN class compared to ORNs of different ORN classes. As our results indicate a cell-autonomous function of Dscam, the actual combination of Dscam isoforms expressed by a single ORN can still rather be stochastic as a coordinated expression between all members of the same ORN class is not necessary.

Surprisingly, the preferential expression of a single Dscam isoform in individual ORNs induces an overgrowth phenotype. Thus, in contrast to the role of Dscam in axon branch segregation^37,46,55^ or dendritic field patterning^38,45^, the signaling activity of Dscam in ORN axons has to be precisely regulated to allow spatially defined convergence. Alternatively, the unique isoform combination expressed on each ORN axon provide a balanced Dscam activity which is sufficient to prevent en passant inter-axonal recognition but can be overcome by external derived signals once the axon enters the target area. The intra-neuronal Dscam signaling could be induced through a binding of the same isoform in *trans* (on adjacent filopodia) or via *cis*-clustering in a single filopodia. Recent structural analyses and *in vitro* studies have shown that homophilic binding requires the assembly of Dscam isoforms in larger molecular clusters^34,63^.

### Developmental mechanisms in olfactory system formation

Sensory maps in *Drosophila* and mammals are characterized by a similar structural organization. In the visual system, photoreceptor neurons projections into the brain visual centers are patterned in a topographic fashion, whereas sensory neurons in the olfactory system segregate according to their OR identity into a discrete synaptic map^14,17^. However, for the formation of the visual and olfactory map there seems to be different developmental control mechanisms employed in flies and mammals. In the mouse visual system, sensory neurons are guided predominantly by gradients of a few secreted factors to the correct location in the target area^64,65^ whereas in flies direct axon-target interactions seems to organize R-cell type specific innervation^66–69^. In the mouse olfactory system, the odorant receptors, expressed on projecting sensory axons, provides unique recognition identities via the regulation of cell-cell signaling molecules^6^. Here we show that olfactory neurons in *Drosophila* also possess distinct axonal identities independent of OR regulation. Similar to the developing mouse olfactory system, self-organization of the sensory map formation through afferent-afferent interaction seems to be the primary control mechanism. In addition, the precise ORN axon targeting requires two independent regulatory mechanisms, one which generates class-specific ORN axon identities (trans cellular signaling) and a second mechanisms which modulates the activity of this axon recognition (cell autonomous signaling). Finally, a certain degree of stochastic expression can be found in both olfactory systems, namely the OR receptor choice in mouse and Dscam isoform expression in flies. On the other hand, in flies, the target region seems to be also involved in the modulation of inter-axonal recognition and we demonstrate unique axon-target recognition in ORN-PN matching. It will be interesting to determine in the future if axon-axon and axon-dendrite interactions are using the same surface recognition code and how this is regulated via Dscam signaling.

### Dscam regulates inter-axonal communication

Interactions between targeting axons is gaining more and more importance as the self-organizing theme underlying olfactory circuit formation^70^. The most compelling evidence comes from mice, where ORNs are able to converge and maintain the gross oderotopic map in the background of an ablated Olfactory bulb^10,71^. Consistent with this, we also found that regulation of interactions between targeting axons is essential for proper Oderotopic map formation in *Drosophila*. In addition to its well-established cell intrinsic role in self neurite repulsion^72^, Dscam regulates inter-axonal interactions between ORNs belonging to same OR class. Though not directly involved, it might cell intrinsically regulate levels of other adhesion molecules which are involved in recognition between ORNs of same OR class. Three observations directly support this hypothesis, (1) formation of ectopic glomeruli outside antennal lobe in *Dscam* mutants, not innervated by PNs, (2) Flybow showing multiple axons in *Dscam* mutant ectopic glomeruli and (3) single cell *Dscam* mutant clones showing no mis-targeting defects. How Dscam achieves regulating inter-axonal interactions is not clear. It might regulate the expression of signaling/adhesion molecules involved in class specific ORN convergence, which itself might depend on a complex combinatorial code. Dscam mediated axonal interactions is one aspect of the multiple processes involved in olfactory circuit formation and will integrate with aspects like Sema/Plexins^30^, Epherins^73^, Teneurins^74^ or DIPs/DPRs^75^ mediated specification of olfactory connectivity or Glia mediated sorting of ORNs as has been shown in Manduca^76^. Interestingly in *Dscam* mutant clones, some mutant ORNs always reach wild type site. This can result from some axons escaping inter-axonal interactions, thus reaching wild type site. Some other possibilities can be a redundancy in the function of various Dscam isoforms^26^ or activity of some recognition molecules in a complex combinatorial code for ORNs targeting may allow some ORNs to target to their wild type sites.

## Methods

### Genetics

Fly stocks were maintained in standard medium at 25 °C unless stated otherwise. Three different *dscam* alleles, reported as null alleles, were used for the analysis: *dscam*^*21*^, *dscam*^*23*^, *dscam*^*33*^^26^. *dscam*^*21*^ allele was used in all experiments in addition to either *dscam*^*23*^ or *dscam*^*33*^.

### Markers for different ORN subclasses and AL neurons

To label ORNs of one single olfactory class we used the following promoters fused to Gal4: Or22a, *Or46a*, *Or47a*^14^, *Or71a*^26^ and *Or88a*^77^. The reporters to visualize axons and synaptic terminals were *UAS-mCD8-GFP*^51^, *UAS-Synaptotagmin-GFP*^78^ and *UAS-rCD2*^79^. To visualize ORNs in mosaics or/and to over-express a single Dscam isoform the enhancer trap line *Gal4-C155* (*elav-Gal4*, all neurons^80^), *SG18*.*1-Gal4* (all ORNs)^81^, *con-Gal4* (lateral ORN classes)^29^, *MT14-Gal4* (olfactory, gustatory and mechano-sensory neurons)^82^, *E132-Gal4* (restricted expression in eye-antennal disc)^83^ and *OK72-Gal4* (antennal lobe glomerulus VM1 and VM4)^27^ were used. The *C753-Gal4* lines are expressed in LNs^84^. Projection neurons were visualized with the enhancer trap lines *GH146-Gal4* and *Mz19-Gal4*^85^. For simultaneous visualization of two ORN classes or target neurons and ORN terminals the *Synaptotagmin-GFP* was directly expressed under Or promotor control^15,27^.

### Genetic mosaics

All of the genetic mosaics were generated using the FRT/FLP system^86^ with various Gal4 drivers (see previous section). For large clones in the antenna and maxillary palps, an *eyless-FLP* insertion on the X chromosome in combination with cell lethal *PCNA* (proliferating cell nuclear antigen, an auxillary protein of DNA polymerase Delta and control eukaryotic DNA replication) on *Dscam*^*+*^ chromosome was used^47,48^. *Eyless-flp* will cause recombination in half of the ORNs generating Dscam^−/−^ ORNs, but not in the central brain cells leaving all other CNS neurons Dscam^−/+^. For small clones and single-cell analysis, an *hsp70-FLP* transgene on the X chromosome was used^87^. To visualize the homozygous mutant ORNs, the MARCM system^51^ with various Gal4 drivers (see previous section) and *FRT42 TubP-Gal80* was used. To visualize ORN subclasses, mosaics were generated in flies of the following genotype: *eyFLP*; *FRT42/FRT42 Gal80; Or-Gal4 UAS-sytGFP* and *eyFLP; FRT42 dscam/FRT42 Gal80; Or-Gal4 UAS-sytGFP*. “Reverse MARCM” genotype was as follows: *eyFLP*; *FRT42 dscam Gal80/FRT42; Or-Gal4 UAS-sytGFP*. To visualize ORN terminals and target cell dendrites, we used the following genotype: *eyFLP; Or::sytGFP FRT42/FRT42 PCNA; C753-Gal4 UAS-CD2* and *eyFLP; FRT42 dscam Or::sytGFP/FRT42 PCNA; C753-Gal4 UAS-CD2*, *eyFLP; GH146-Gal4FRT42/FRT42 PCNA; Or::sytGFP UAS-CD2*, *eyFLP; FRT42 dscam GH146-Gal4/FRT42 PCNA; Or::sytGFP UAS-CD2*. Double labelling of two ORN single classes one Or::sytGFP fusion construct and one Or-Gal4 UAS-CD2 line is used as follows: *eyFLP UAS-CD2; FRT42 Or::sytGFP/FRT42 PCNA; Or-Gal4 UAS-CD2* and *eyFLP UAS-CD2; FRT42 dscam Or::sytGFP/FRT42 PCNA; Or-Gal4 UAS-CD2*. To label the projection of single neurons in wild type and with over-expression of a single *dscam* isoform, this genotype was used: *hsFLP*, *elav-Gal4*, *UAS-mCD8GFP; FRT42 Gal80/FRT42* and *hsFLP*, *elav-Gal4*, *UAS-mCD8GFP; FRT42 Gal80/FRT42 UAS-dscam*^*17*.*2-7*^. Single-cell clones were obtained by heat shocking late third-instar larvae (10 min at 37 °C). Developmental studies with the marker *elav-Gal4* were performed using pupae of the following genotypes: *eyFLP; FRT42/FRT42 Gal80; elav-Gal4 UAS-CD8GFP* and *eyFLP; dscam FRT42/FRT42 Gal80; elav-Gal4 UAS-CD8GFP* and *eyFLP elav-Gal4; FRT42 Gal80/FRT42 UAS-dscam*^*17*.*2-7*^. For all these experiments, we used *Dscam*^*21*^ and *Dscam*^*33*^ in parallel^26^.

### Immunohistology

Primary antibodies used for immunohistochemistry were: rat anti-N-Cadherin extracellular domain (DN-Ex #8; 1:20^88^, 1997, DSHB); rabbit anti-GFP (1:1000; Molecular Probes); and mouse anti-CD2 (1:1000; Molecular Probes). Secondary antibodies used were as follows (all 1:300): goat anti-rabbit F(ab)′ fragment coupled to Alexa 488 (Molecular Probes), goat anti-mouse F(ab)′ fragment coupled to Alexa 568 (Molecular Probes), goat anti-mouse F(ab)′ fragment coupled to Alexa 568 highly cross-absorbed (Molecular Probes), goat anti-rat F(ab)′ fragment coupled to Alexa 568 (Molecular Probes), goat anti-rat F(ab)′ fragment coupled to Alexa 647 (Molecular Probes) and Toto-3 (1:2000, Molecular Probes). Immunostaining of brains of adult flies and pupae were carried out essentially as described previously^89^ with the following exceptions: (1) adult brains were fixed in 2% PFA for 90 min, and (2) for the dissection of the pupal brains, the pupal cases were open, 2% PFA was added, and the brains were allowed to fix for 10 min before further dissection in 2% PFA. The overall time of fixation in 2% PFA was 90 min. Fluorescent samples were analyzed using a Zeiss Meta510 confocal microscope.

### Image processing

The majority of the images were processed using Fiji®^90^. For single cell clones, the stacks of confocal images were first 3D rendered in Imaris®. Then with the “Surface” tool, the innervation site of the axon in antennal lobe and the cell bodies in antenna were reconstructed by thresholding intensity. Then the axonal fibers of the neuron were reconstructed using the tool “Filaments”. This was carried out manually because of the low intensity of GFP in the axons and the high background noise.

## Supplementary information

Supplementary Information

## Data Availability

All the data generated and analyzed during this study are included in this published article and its supplementary information files.
