# Supplementary material for: Inter-axonal recognition organizes Drosophila olfactory map formation
Source: Sci Rep. 2019 Aug 9;9:11554. doi: 10.1038/s41598-019-47924-9 (PMC6689066; doi:10.1038/s41598-019-47924-9)
Supplement: Supplementary file 1 — Supplementary Information [file 41598_2019_47924_MOESM1_ESM.pdf]

Supplementary Information

Inter-axonal recognition organizes *Drosophila* olfactory map formation

Gaurav Goyal*[1], Ariane Zierau[2], Marc Lattemann[2], Beate Bergkirchner[1], Dominik Javorski[1], Rashmit Kaur[1] and Thomas Hummel[1,2]

[1]Department for Neurobiology, University of Vienna, Althanstrasse 14, 1090, Vienna, Austria

[2]Institut für Neuro- und Verhaltensbiology, Universität Münster, Badestr. 9, D-48149 Münster, Germany

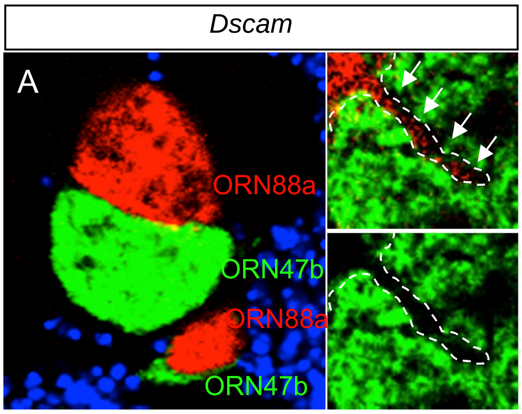

Supplementary Fig. S1: *Dscam* mutant axons converge class-specifically in ectopic spots

Neighbouring projecting ORN classes 47b and 88a show distinct boundaries in *Dscam* mutants. *eyflp UAS-CD2; FRT42 Dscam 47b::sytGFP/FRT42 PCNA; 88a-Gal4 UAS-CD2.* Green: syt-GFP; red: ratCD2; blue: Toto3

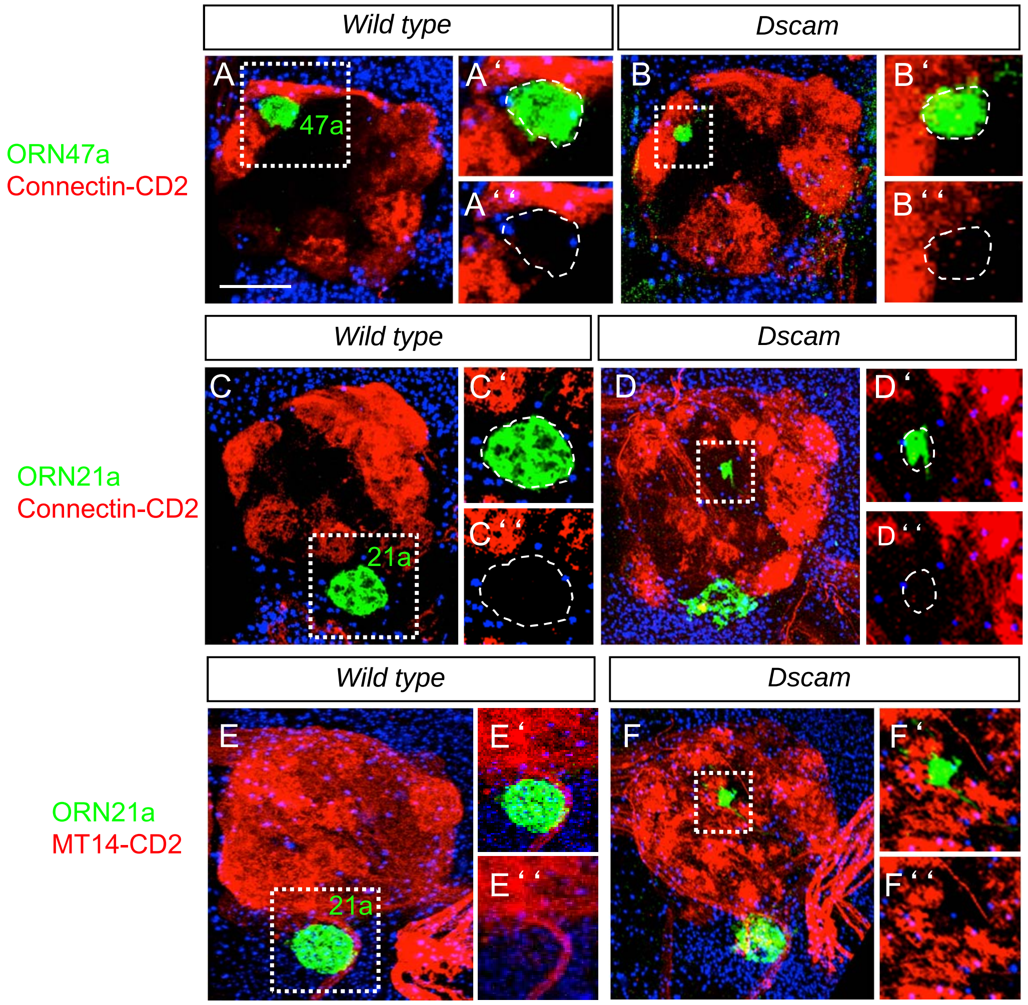

Supplementary Fig. S2: *Dscam* mutant axons converge class-specifically in ectopic spots

Connectin-Gal4 is expressed in multiple ORN classes but not in ORN47a (A, A', A") and ORN21a (C,C',C"). Ectopic ORN47a (B,B',B") and ORN21a (D,D',D") spots are never innervated by *con*-positive axons. *MT14-Gal4* labels a subset of ORN classes but not the ORN class 21a (E,E',E"). *Dscam* mutant ORN21a axons create ectopic spots inside the AL, but they never converge with *MT14* positive axons (F,F',F"). *Genotype: (A) eyflp UAS-CD2; FRT42 47a::sytGFP/FRT42 PCNA; con-Gal4 UAS-CD2, (B) eyflp UAS-CD2; FRT42 Dscam 47a::sytGFP/FRT42 PCNA; con-Gal4 UAS-CD2, (C) eyflp UAS-CD2; FRT42 21a::sytGFP/FRT42 PCNA; con-Gal4 UAS-CD2, (D) eyflp UAS-CD2; FRT42 Dscam 21a::sytGFP/FRT42 PCNA; con-Gal4 UAS-CD2, (E) eyflp UAS-CD2; FRT42 21a::sytGFP/FRT42 PCNA; MT14-Gal4 UAS-CD2, (F) eyflp UAS-CD2; FRT42 Dscam 21a::sytGFP /FRT42 PCNA; MT14-Gal4 UAS-CD2.* Green: syt-GFP; red: ratCD2; blue: Toto3, Scale bar: 25μm

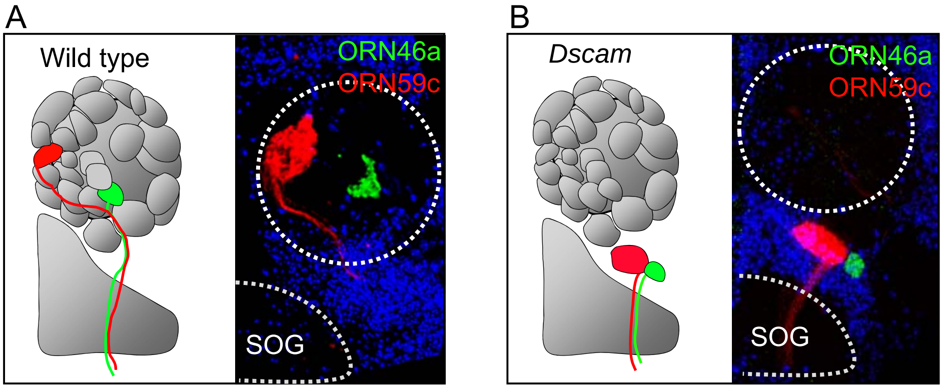

C

	Or47a	Or47b	Or46a	Or21a
Or47a		✓		
Or22a	✓			
Or21a	✓	✓		
Or88a	✓	✓		
Or59c	✓	✓	✓	
Or42a	✓	✓		
Or71a			✓	
Con-Gal4	✓			✓
MT14-Gal4			✓	✓

Supplementary Fig. S3: class-specific axon-sorting outside the AL of maxillary palp ORN classes

ORN classes from the maxillary palp, which innervating in wild type distant glomeruli (A) converge in *Dscam* mutant in close neighbourhood but class-specific (B). C) Table compiling all the combination of OR classes/broad Gal4 checked for analyzing class specificity of ORN targeting. In none of the cases any ORN class mixing was observed. SOG: suboesophagial ganglion..*Genotype: (A) eyflp UAS-CD2; FRT42/FRT42 PCNA; 46a::sytGFP 59c-Gal4 UAS-CD2. (B) eyflp UAS-CD2; FRT42 Dscam/FRT42 PCNA; 46a::sytGFP 59c-Gal4 UAS-CD2.* Green: sytGFP, red: ratCD2, blue: Toto3

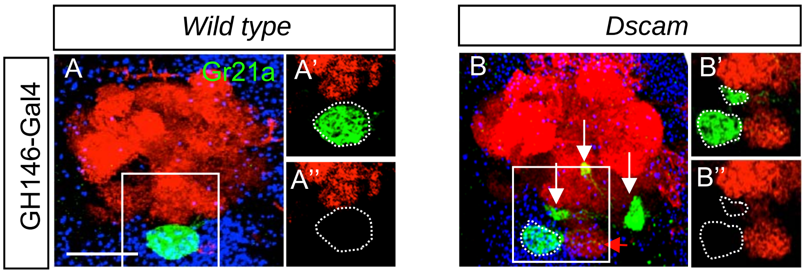

Supplementary Fig. S4: ORN-PN matching identities remain in Dscam mutants.

A-A'') Wild type Gr21a ORNs do not make contacts with GH146-positive PNs. B-B'') In Dscam mutants also, ectopically targeting Gr21a axons do not make contacts with GH146 positive PNs.

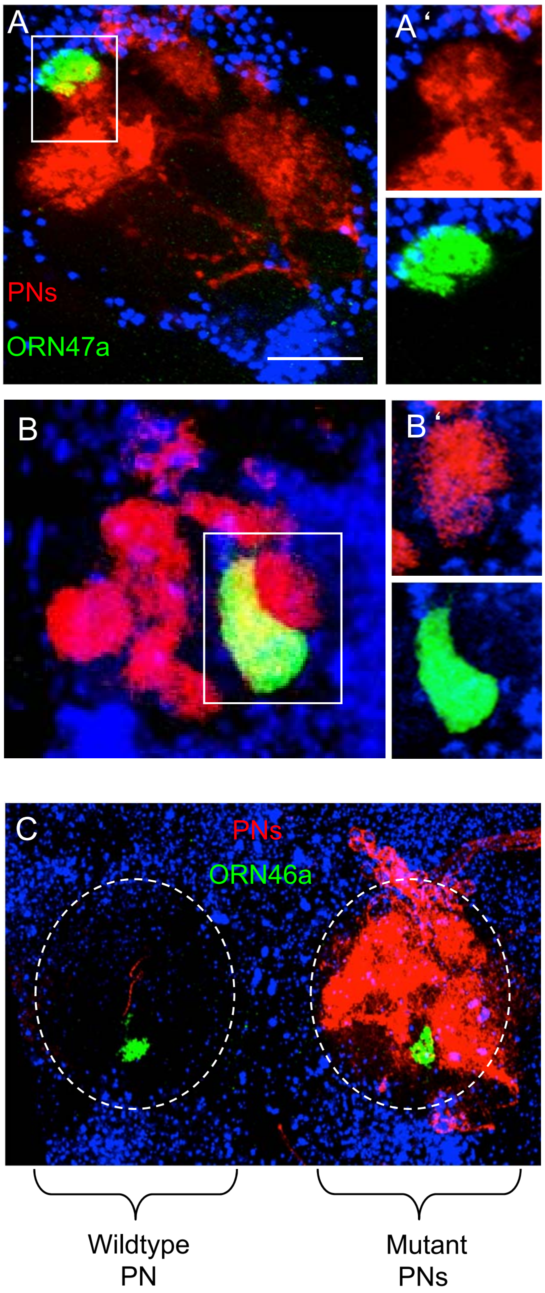

Supplementary Fig. S5: *Dscam* mutant PNs do not alter glomerulus formation of ORNs

Induction of *Dscam* mutant PN MARCM clones does not lead to changes of the targeting of ORN axons of different classes. (A) ORN47a. (B) ORN47b. (C) ORN46a with internal control: *hsFlp* induced clones only in the right AL hemisphere; the PNs in the left hemisphere are wild type. *Genotype: hsflp UAS-CD2; FRT42 Dscam OR::sytGFP/FRT42 Gal80; GH146-Gal4 UAS-CD2.* Green: sytGFP, red: ratCD2, blue: (A-B, D) Toto3, (C) Ncad. Scale bar: 25µm

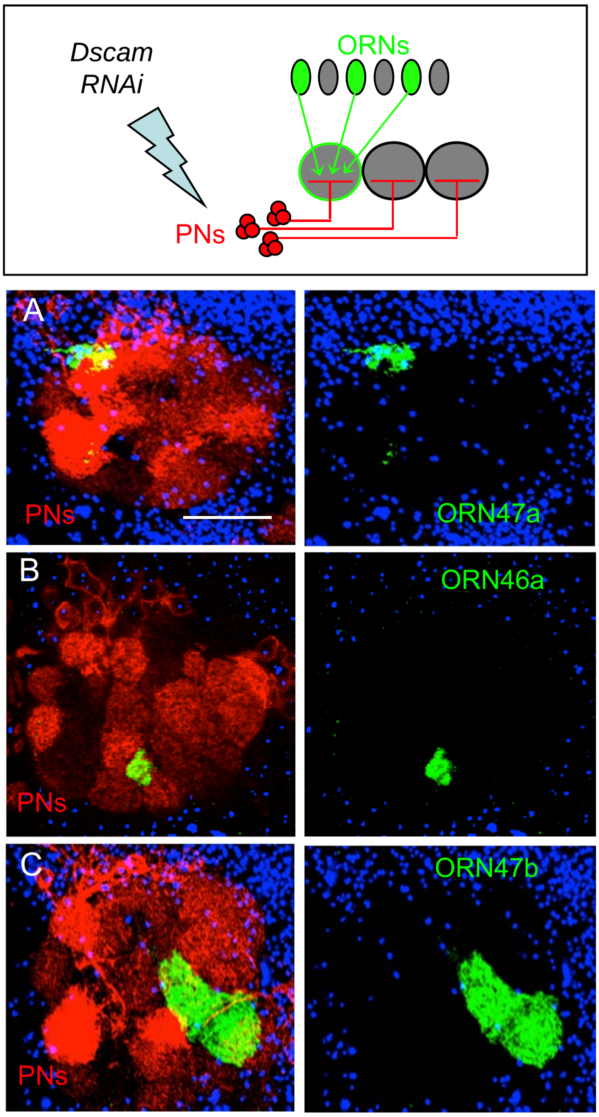

Supplementary Fig. S6: Glomerulus formation of ORNs is not influenced by PNs with *Dscam* knock-down

Dscam^{RNAi} expression in PNs does not affect glomerulus formation of ORN axons of different ORN classes: (A) ORN47a, (B) ORN46a, (C) ORN47b. *Genotype: OR::sytGFP; GH146-Gal4, UAS-CD2/ UAS-Dscam^{RNAi}*. Green: sytGFP, red: ratCD2, blue: Toto3. Scale bar: 25µm

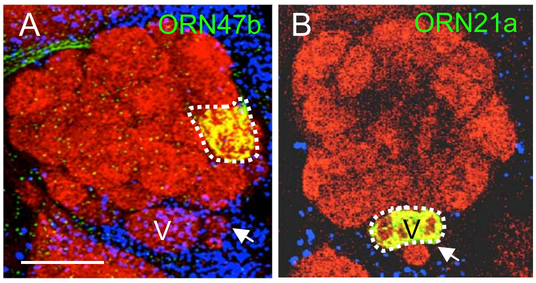

Supplementary Fig. S7: reverseMARCM experiments reveal no mistargeting of wild type ORN axons in *Dscam* mutant background.

Homozygous wild type ORN axons of several classes ((A) ORN47b, (B) ORN21a) always reach their wild type glomerulus even in *Dscam* mutant background, indicated by the ectopic spots outside the AL, near the V glomerulus (arrows in A, B). *Genotype: eyflp; FRT42 Dscam Gal80/FRT42; OR-Gal4 UAS-sytGFP.* Green: sytGFP, red: N-cad, blue: Toto3. Scale bar: 25μm

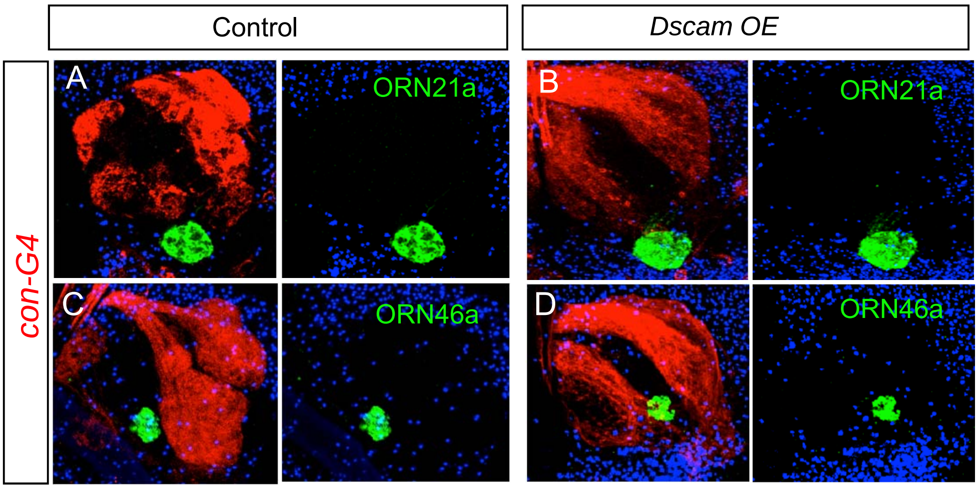

Supplementary Fig. S8: Over-expression of Dscam does not affect targeting of neighboring projecting ORN classes.

Over-expression of Dscam with *con-Gal4* does not disturb targeting of ORN axons, which are outside of the over-expression domain (A, B) ORN21a; (C, D) ORN46a. *Genotype: (A, C) OR::sytGFP; con-Gal4 UAS-CD2. (B, D) OR::sytGFP; con-Gal4 UAS-CD2 / UAS-Dscam*$^{17.2-7}$. Green: sytGFP, red: ratCD2, blue: Toto3. Scale bar: 25µm

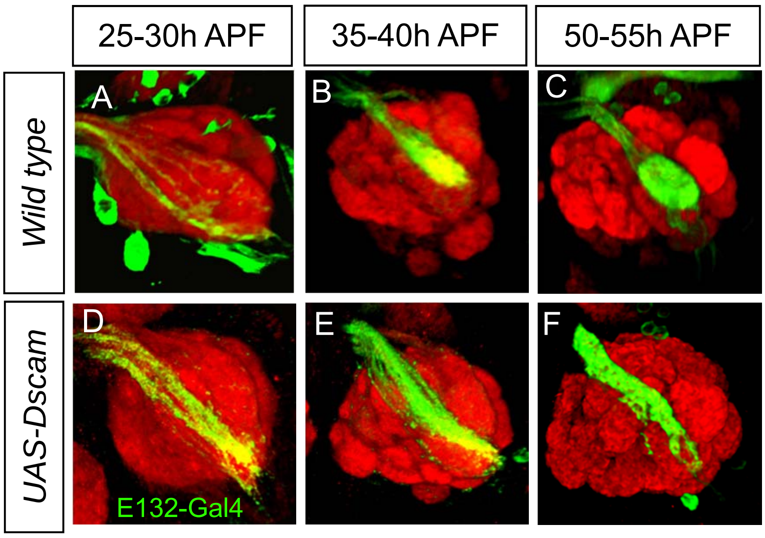

Supplementary Fig. S9: Over-expression of Dscam in a single ORN-class reveals loss of convergence in early pupal development.

Over-expression of a single Dscam isoform in *E132-Gal4* during pupal development shows a disruption of axon convergence early in pupal development. *Genotype: (A-C) eyflp E132-Gal4 UAS-CD8GFP; FRT42 Gal80/FRT42. (D-F) eyflp E132-Gal4 UAS-CD8GFP; FRT42 Gal80/FRT42; UAS-Dscam$^{17.2\text{-}7}$.* Green: CD8-GFP, red: N-cad, blue: Toto3
